# Objective Pain Assessment Using Deep Learning Through EEG-Based Brain–Computer Interfaces

**DOI:** 10.3390/biology14020210

**Published:** 2025-02-17

**Authors:** Abeer Al-Nafjan, Hadeel Alshehri, Mashael Aldayel

**Affiliations:** 1 Computer Science Department, College of Computer and Information Sciences, Imam Mohammad Ibn Saud Islamic University (IMSIU), Riyadh 11432, Saudi Arabia; 2 Information Technology Department, College of Computer and Information Sciences, King Saud University, Riyadh 11543, Saudi Arabia

**Keywords:** brain–computer interface (BCI), electroencephalography (EEG), pain assessment, artificial intelligence, deep learning

## Abstract

Objective pain measurements are essential in clinical settings for determining effective treatment strategies. This study aims to utilize brain–computer interface technology for reliable pain classification and detection. We developed an electroencephalography-based pain detection system comprising two main components: (1) pain/no-pain detection and (2) pain severity classification across three levels: low, moderate, and high. Deep learning models, including convolutional neural networks and recurrent neural networks, were employed to classify the wavelet features extracted through time–frequency domain analysis. Furthermore, we compared the performance of our system against conventional machine learning models, such as support vector machines and random forest classifiers. Our deep learning approach outperformed the baseline models, achieving accuracies of 91.84% for pain/no-pain detection and 87.94% for pain severity classification, respectively.

## Simple Summary

This study addresses the need for accurate pain assessment in clinical settings, especially for patients who cannot communicate their pain levels. We developed a system using brain–computer interface (BCI) technology to automate pain detection through electroencephalography (EEG). Our system identifies whether a person is in pain and classifies its severity into low, moderate, and high levels. Using advanced deep learning models, we achieved high accuracy rates of 91.84% for pain detection and 87.94% for pain severity classification. By comparing our results to traditional methods, we demonstrated that our approach is more effective. This research has the potential to improve pain assessment in healthcare, leading to better treatment strategies for patients.

## 1. Introduction

Pain assessment is a challenging task. Subjective pain assessment relies on individuals’ self-reports, often using scales or questionnaires. Modern clinical methods frequently require healthcare professionals to interview patients to determine their pain levels. These approaches can be difficult for individuals who cannot communicate verbally, highlighting the urgent need for effective and objective pain detection methods [1]. In contrast, objective pain assessment utilizes measurable physiological indicators, such as EEG readings, to evaluate pain, thereby minimizing reliance on self-reported experiences.

A brain–computer interface (BCI) is a communication system that links the brain to external devices, acting as a medium of communication between humans and computers [2]. BCIs translate brain activity signals into the necessary output [3]. BCIs have demonstrated potential in various healthcare domains, including stroke rehabilitation, mental health evaluation, and communication restoration [4]. By analyzing brain activity patterns, BCI systems can provide insights into cognitive processes, attention levels, and emotional states. This information can be used to optimize training programs, assess cognitive workload, and detect signs of mental fatigue [5].

In recent years, researchers in neuroscience and neuroimaging have explored the use of BCIs for objective pain detection [6,7]. In this study, we investigate the potential of BCI technology as a reliable tool for measuring and assessing pain. Specifically, we implement an electroencephalography (EEG)-based pain detection system using BCI technology. Previous studies have applied EEG data to pain detection and severity classification.

The present study automates the current pain self-assessment procedure and transforms it into an automated pain detection process. Therefore, the significance of this study lies in its potential to push the boundaries of pain detection methodologies, thereby improving the accuracy and efficiency of pain assessment.

In this study, we aim to investigate the potential of BCI technology as a reliable tool for measuring and assessing pain. Specifically, we aim to implement an EEG-based pain detection system using BCI methodology. EEG data have been utilized to investigate existing research on pain detection and pain intensity classification. By reviewing the current body of knowledge on pain assessment, this study uncovers valuable insights into the relationship between neural activity patterns, as captured by EEG signals, and the subjective experience of pain. Through rigorous analysis and experimentation, we develop robust algorithms and methodologies that can accurately detect and quantify pain levels objectively.

This study aims to detect pain based on pain perception using EEG-based BCI technology. This research aims to automate the current pain self-assessment procedure and transform it into an automated pain detection process. Therefore, the significance of this research lies in its potential to push the boundaries of pain detection methodology, thereby enhancing the accuracy and efficiency of pain assessment.

The primary research question addressed in this study is the following: can systems based on brain–computer interfaces (BCIs) accurately assess pain levels using objective measures of the affective state of the brain? We hypothesize that using deep learning models will significantly improve the accuracy of pain assessment from EEG signals. EEG-based BCI systems can accurately differentiate between affective states during pain assessment. This work aims to explore the potential of EEG-based BCI systems for distinguishing between different affective states related to pain.

The remainder of this paper is structured as follows. The background is presented in Section 2. Section 3 explains the materials and methods of the proposed system. Section 4 presents the results. Section 5 presents a discussion. Section 6 concludes the study.

## 2. Background

Machine learning (ML) has emerged as a key branch of artificial intelligence (AI), because it offers a diverse range of symbolic and statistical approaches for analyzing and interpreting neural data. ML empowers systems to learn and refine themselves through experience by exploiting computational models to generate predictions or decisions without requiring explicit programming. In the context of BCIs, ML plays a critical role in various applications, revolutionizing the field by improving the diagnosis of various diseases and sleep disorders, predicting epileptic seizures, and facilitating rehabilitation [8].

Conventionally, BCI systems have several limitations in terms of learning, understanding, and interpreting complex brain activities. However, the adoption of ML in BCI systems has addressed these challenges. ML algorithms excel at feature learning and complex pattern recognition, thereby improving the efficacy of BCI stages, particularly signal feature extraction and classification. Consequently, ML empowers BCI systems to perform tasks with higher precision and utility [8].

ML has become an indispensable tool in the domain of BCIs. ML enables the analysis, interpretation, and utilization of complex brain signals. The integration of ML algorithms allows BCI systems to overcome limitations, improve feature extraction, and realize breakthroughs in pattern recognition. Through the application of ML techniques, BCIs are poised to significantly improve our understanding of the brain and the quality of human–computer interactions [9].

Deep learning (DL) is a specialized field within ML that uses extensive data and multiple neural network layers to enhance its learning capabilities. In essence, DL can be regarded as a powerful model within the ML domain that is inspired by the intricate workings of the human brain. In addition, both DL and ML are subsets of AI [10]. DL can uncover complex patterns and relationships within data, resulting in improved accuracy and decision making for AI models. DL is effective in areas such as natural language processing (text), automatic speech recognition (audio), computer vision (image), and intrusion detection [10].

**Figure 1:**
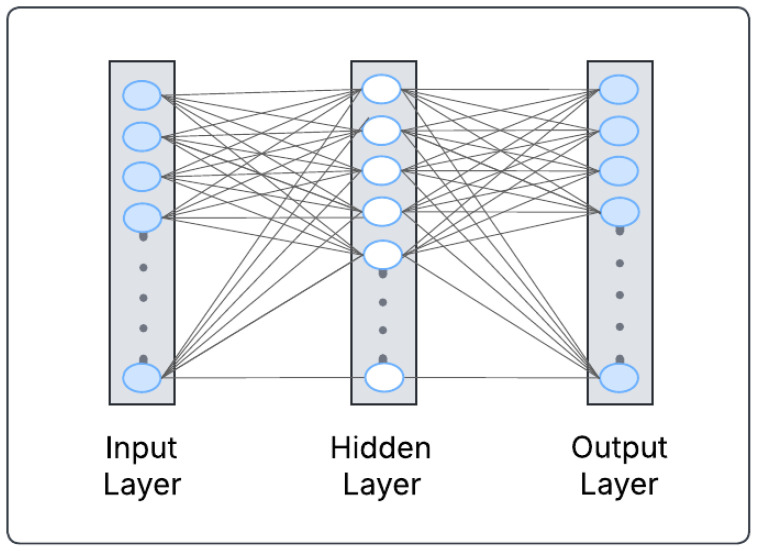
Neural network.

The concept of DL symbolizes the idea of successive layers of representations. This means that the depth of a DL model reflects the number of layers that contribute to the model [10]. Modern DL architectures typically comprise tens or even hundreds of successive layers of representations, all of which are learned automatically from training data. In addition, DL comprises neural networks, which are composed of layers stacked on top of each other and can be trained to learn [10]. Figure 1 illustrates the general architecture of DL.

The two most common types of DL network architectures are convolutional neural networks (CNNs) and recurrent neural networks (RNNs). CNNs constitute the most popular DL models in BCI research, because they can be used to exploit the latent spatial dependencies among brain input signals, such as fMRI images and spontaneous EEG. CNNs possess several salient features that make them well suited to computer vision applications, including a regularized structure, good spatial locality, and translation in variance. These properties enable CNNs to effectively discover latent spatial information, making them ideal for tasks such as image recognition and object searching.

**Figure 2:**
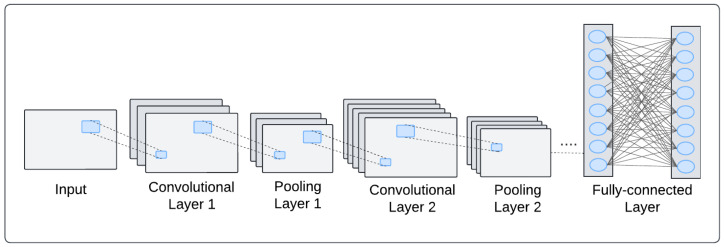
Convolutional neural network.

In BCIs, CNNs have been employed to capture the distinctive dependencies among patterns associated with different brain signals. The standard architecture of CNNs comprises three stacked layers: convolutional, pooling, and fully connected layers (Figure 2).

The convolutional layer forms the core block of a CNN. It contains a set of filters that convolve the input data. This is then followed by a nonlinear transformation of the data to extract geographical features. The pooling layer aims to reduce the spatial size of the features. The fully connected layer, similar to a basic neural network, connects each node to all activation nodes in the previous layer [11].

RNNs comprise a specific subclass of discriminative DL models specifically designed to capture temporal dependencies among input data. RNNs achieve this by incorporating recurrent connections, which can be considered cycles within a network of nodes. This cyclical structure allows RNNs to process sequential data effectively. Furthermore, RNNs are specifically designed to handle time series or sequential data. They incorporate the concept of “memory”, which allows them to store the states or information of previous inputs to predict or generate the next output in the sequence. RNNs have a long history and originated as brain models popularized by cognitive scientists and subsequently adopted as practical modeling tools. They are well suited to tasks such as natural language processing and time series forecasting [12].

Two typical RNN architectures have garnered significant attention and achieved remarkable success: long short-term memory (LSTM) and gated recurrent units (GRUs) [11].

**Figure 3:**
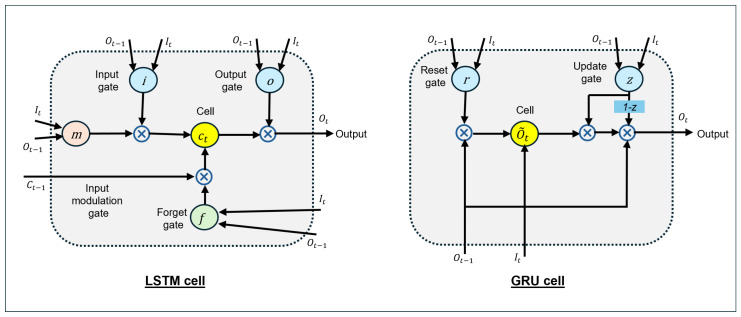
LSTM and GRU cell structures. (**left**) LTSM; (**right**) GRU.

- LSTM controls data flow using four gates: the input gate, output gate, forget gate, and input modulation gate. The LSTM cell receives three inputs and exports two outputs, as illustrated in Figure 3. The inputs include the hidden state of the previous time ($c_{t}-1$), the output of the previous time ($O_{t}-1$), and the input at the current time ($I_{t}$). The output of the cell serves as the input to the next cell, which is the hidden state and output of the current time [11].
- GRU is another widely used RNN architecture. Similar to LSTM, GRU exploits information from the past. GRU receives temporal information only from the output at time t−1; unlike LSTM, it does not require hidden states. GRU has two inputs ($I_{t}$ and $O_{t}-1$) and one output ($O_{t}$), and it contains two gates: the reset gate *r* and the update gate *z* [11]. As shown in Figure 3, the input and output mapping can be described as follows:
  [It,Ot−1→Ot]

LSTM and GRU are two widely used RNN architectures that have demonstrated superior performance in various applications, including BCIs. Although both LSTM and GRU are very similar and exhibit comparable performance, as reported in the literature [11], the choice between the two algorithms may depend on specific tasks and the available computational resources.

## 3. Materials and Methods

**Figure 4:**
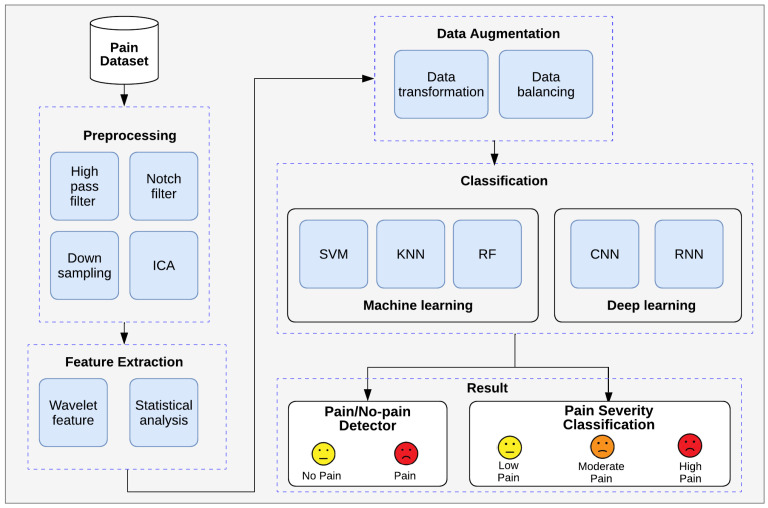
Framework for our proposed EEG-based pain detection system.

This section presents the proposed system framework for pain detection employing an EEG-based BCI system. As shown in Figure 4, the proposed framework comprises four key stages: signal processing, feature extraction, data augmentation, and classification.

The signal processing stage involves preprocessing EEG signals through various filters, such as bandpass filters, notch filters, and downsampling techniques. To further enhance data quality and reduce noise artifacts, independent component analysis (ICA) is implemented. Feature extraction employs wavelet transformation and statistical analysis to extract pain-relevant features from preprocessed EEG signals. Wavelet analysis facilitates the identification of time–frequency characteristics associated with pain patterns in brainwaves.

Data augmentation techniques, including the synthetic minority oversampling technique (SMOTE), solve class imbalance problems, guaranteeing more representative datasets for model training. Finally, the classification stage employs both ML and DL approaches to train models capable of accurately classifying pain severity based on the extracted features.

This section further details the selected benchmark dataset and the feature extraction methods specifically designed to identify pain-related features in EEG signals. Using the proposed framework and selected dataset, we investigated the effect of data augmentation on the performance of pain assessment systems. Subsequent sections explain each component of the proposed system framework in detail.

### 3.1. EEG Dataset

This subsection introduces the benchmark dataset applied to the proposed EEG-based pain detection system. The selected dataset, named “Brain Mediators for Pain”, was published by Tiemann et al. [13] and consists of EEG signals collected from healthy participant populations in Germany.

The dataset encompasses data from 52 right-handed individuals (48% female; 52% male). Each participant was subjected to four experimental conditions: perception, motor, autonomic, and combined. These distinct conditions were employed in the experimental paradigm to elicit pain responses and corresponding physiological data.

The details of the pain measurements for each condition are presented in Table 1.

#### 3.1.1. Study Group

The study involved a total of 51 healthy participants, comprising a diverse sample in terms of sex and age. Among them, 26 participants identified as female (sex code 2) and 25 as male (sex code 1). The ages of the participants ranged from 20 to 37 years, with a mean age of approximately 27 years. Each participant’s pain threshold, measured in millijoules (mJ), varied significantly, indicating individual differences in pain sensitivity. The pain thresholds ranged from a low of 147 mJ to a high of 660 mJ, highlighting a broad spectrum of pain response within the healthy population. Importantly, all participants reported no history of chronic pain, ensuring that the findings were reflective of typical pain processing and responses rather than influenced by prior pain experiences. This demographic information provides a comprehensive context for interpreting the experimental results related to pain perception and response.

#### 3.1.2. Pain Scale

This research investigates the effectiveness of two classification formulations for EEG-based pain detection: pain/no-pain detector and pain severity classification. Class labels are assigned to the EEG data to facilitate more manageable and interpretable representations. The pain rating thresholds are mapped into the corresponding class labels, resulting in either a pain/no-pain or pain severity classification scheme.

**Pain/No-pain Detector** This study aims to categorize pain into two mutually exclusive classes: “no pain” and “pain”. This classification is achieved by implementing a thresholding approach, in which pain scores equal to or lower than 5 are assigned the label “no pain” while scores exceeding 5 are labeled as “pain”. This binary categorization effectively differentiates between pain and no-pain detection. The 5 threshold is often used to indicate the transition from mild discomfort to significant pain, which aligns with established practices in pain research [14,15].

**Pain Severity Classification** A discrete labeling scheme is employed to categorize pain scores into three distinct classes: “low pain”, “moderate pain”, and “high pain”. This mapping is achieved through a rule-based approach. Scores equal to or less than 3 are assigned the label “low pain”. Scores between 4 and 6 (inclusive) are classified as “moderate pain”. Finally, scores exceeding 6 are designated as “high pain” [14,15].

#### 3.1.3. Dataset Exploration

This section details the exploration and identification processes of the pain EEG dataset.

**Figure 5:**
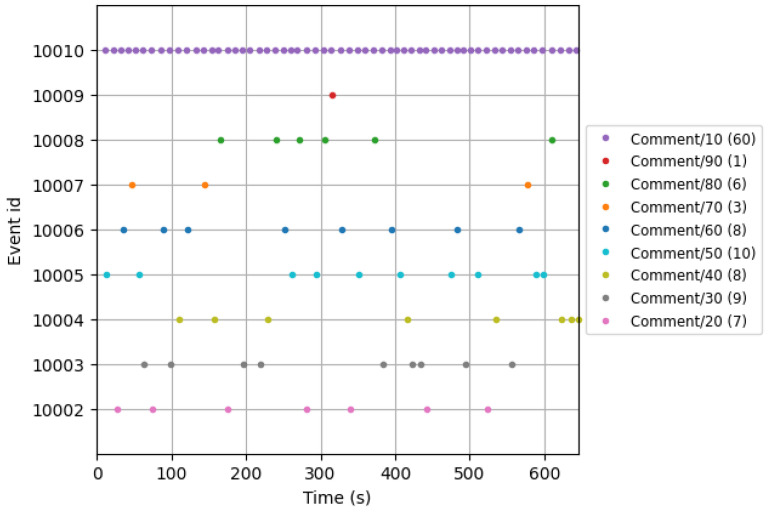
Data from a participant’s experiment; the notation Comment/10(60) indicates that 10 represents the threshold, while 60 reflects the total number of times pain severity was perceived. Specifically, a pain severity rating of 5 was recorded 10 times, and a rating of 8 was recorded 6 times.

Figure 5 illustrates an experimental trial conducted on a participant. The color-coded events correspond to distinct pain thresholds. Specifically, event ID “10010” represents a pain threshold of 10, and event ID “10005” represents a pain threshold of 5. The epochs throughout the experiment are represented by the plotted dots.

The pain EEG dataset was recorded using the EasyCap device equipment sourced from EasyCap GmbH, located in Herrsching, Germany. BrainVision software version (1.27.0001) was used. During the recording session, participants wore an EasyCap positioned on their heads and had ice-induced pain stimuli applied to their hands. The participants then concurrently provided pain severity ratings on a scale of 1 to 10. The dataset consists of three distinct files: a header file with a .vhdr extension, a data file with a .eeg extension, and an event marker file with a .vmrk extension.

We employed the functionalities offered by the MNE library using python programming language to more comprehensively explore the dataset. This enabled us to gain comprehensive insights into the data, ultimately allowing for the effective analysis and interpretation of neural responses associated with pain perception. To gain insights into the dataset, we employed several functions and attributes from MNE library such as info attribute; we utilized this to explore the dataset characteristics. This attribute revealed details such as the number of channels and sampling frequency. The data consisted of 68 EEG channels and 1 miscellaneous channel, indicating a comprehensive electrode montage. Notably, the sampling frequency of 1000 Hz provided high temporal resolution and captured rapid changes in the EEG signal. Additionally, we employed the set_montage function within MNE-Python to define channel locations. However, discrepancies were observed between the default montage’s channel positions and the expected positions. The EasyCap device company was contacted to address this inconsistency and acquire precise channel location information in terms of X, Y, and Z coordinates. A custom montage was then generated by passing these acquired channel locations (stored as the ch_pos attribute) to the make_dig_montage() function in MNE-Python. This custom montage was subsequently applied to the raw EEG data using the set_montage() function.

Furthermore, the Epochs() function from MNE was employed to structure the extracted data into epochs. This function segmented the continuous EEG data based on the extracted event markers. The resulting epochs with duration from 8 to 12 s represented the EEG activity during specific time windows corresponding to each pain level. The resulting data then took the form of events and epochs—events denoted distinct pain levels and epochs represented the entire duration corresponding to each pain level.

### 3.2. Signal Preprocessing

This section details the signal preprocessing techniques employed to improve the quality of pain-related EEG signals. Based on a literature review of pain detection using EEG [16,17], four distinct filtering operations were applied: high-pass filtering, notch filtering, downsampling, and ICA.

1. A high-pass filter was implemented to attenuate low-frequency artifacts such as baseline drift. This approach is in agreement with previous studies using the same dataset [16,17], which have demonstrated the efficacy of high-pass filtering for removing low-frequency components from EEG data. To ensure consistency and facilitate the comparison of the results, we adopted the same high-pass filter with a cutoff frequency of 1 Hz employed in the aforementioned studies. This approach aimed to suppress low-frequency noise while preserving pain-relevant higher-frequency information in EEG signals. Additionally, we conducted preliminary analyses to evaluate the impact of different cutoff frequencies (e.g., 0.5 Hz and 2 Hz) on the signal characteristics and found that a cutoff of 1 Hz provided an optimal balance between removing artifacts and retaining crucial information for pain assessment. This approach aligns with best practices in EEG signal processing, ensuring that our results are reliable and comparable to existing literature.
2. A notch filter was subsequently applied to eliminate specific narrowband noise, particularly power line interference. This strategy is in line with recent studies highlighting the importance of notch filtering in EEG analysis on similar datasets [16,17]. In our experiment, we applied the notch filter at 50 Hz, mirroring the approach adopted in the referenced studies. This process effectively removed power line interference, thereby improving signal quality and the accuracy of subsequent analyses. Additionally, we conducted preliminary analyses to evaluate the impact of different notch filter settings (e.g., 45 Hz and 55 Hz) on the signal characteristics. Our findings indicated that applying the notch filter at 50 Hz effectively attenuated power line noise while preserving essential EEG information relevant to pain assessment.
3. The EEG signals were downsampled to a sampling rate of 500 Hz using an automated approach. Downsampling reduces computational demands while retaining essential information for further analysis. In the experiment, the sampling rate was reduced from 1000 to 500 Hz. This technique effectively minimizes the dataset size without incurring significant information loss. To mitigate potential information loss from minimizing the dataset size, we conducted a preliminary analysis comparing the pain-related features extracted from both sampling rates. Our findings indicated that the essential characteristics of the signals were preserved, ensuring that the downsampled data remained suitable for subsequent analyses. Nonetheless, we acknowledge the importance of carefully considering the implications of this reduction in sampling rate and have taken steps to validate the integrity of the pain-related features during our analysis.
4. ICA was employed to remove residual noise and artifacts from the preprocessed EEG signals. ICA is a well-established signal processing tool and is recognized for its effectiveness in artifact removal, denoising, and signal enhancement. The Infomax algorithm for ICA was applied to the EEG data to identify and extract independent components. The analysis was conducted on data from 62 channels, resulting in the extraction of 62 independent components. This approach allows us to retain as much spatial information as possible while identifying and isolating artifacts. The extracted components were then evaluated for the presence of noise, and any components deemed to represent artifacts were removed prior to further analysis. To ensure the robustness of our results, ICA was applied consistently across all subjects, utilizing a dataset of 329 matching events without baseline correction.

After the preprocessing stage, the EEG signals underwent quality enhancement that rendered them suitable for subsequent feature extraction. This transformation facilitated the conversion of raw EEG signals into a more informative representation.

### 3.3. Feature Extraction

This section explains the feature extraction process, which contributes to the extraction of informative patterns from EEG signals. Based on the literature review, the importance of leveraging time–frequency characteristics for pain detection in EEG signals has been established [18,19,20,21]. This study employed wavelet transform as a feature extraction technique due to its effectiveness in identifying pain-related patterns within the time–frequency domain of EEG signals [22]. Wavelet transform decomposes EEG data into distinct frequency components using discrete wavelet transform (DWT), enabling separate analysis of each frequency band and identification of pain-related features within these bands. In particular, we focus on EEG patterns such as increased alpha or beta activity, which have been correlated with pain perception in previous studies.

**Figure 6:**
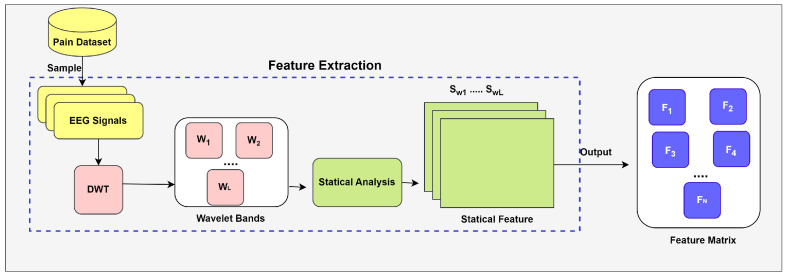
Feature extraction process.

Although several studies discussed in the literature review employ various statistical methods for feature extraction [20,23,24], this study explores the potential of using multiple statistical measures for each wavelet coefficient. Specifically, we compute these statistical measures to analyze the distribution of information within each frequency band of the decomposed EEG signal, as shown in Figure 6 where *W* represents a wavelet band, DWT is a wavelet coefficient generated by wavelet transformation, *L* represents the number of wavelet bands, $S_{w}$ denotes a statistical feature calculated for each wavelet coefficient, *F* represents an individual feature vector, and *N* represents the number of samples.

#### 3.3.1. Wavelet Transform

Wavelet transform is a valuable mathematical technique for decomposing a signal into its different frequency components. This technique facilitates the analysis of signals in the time–frequency domain, enabling the extraction of features localized in both time and frequency. Within the proposed system, DWT was employed to decompose the EEG signals into wavelet coefficients. DWT is a multiresolution analysis technique that decomposes signals into various frequency bands with corresponding resolutions. This decomposition produces wavelet coefficients representing signals at different scales, allowing for the capture of both global and localized features in EEG data [22]. We applied the wavelet transform using the Daubechies 4 (db4) wavelet for time–frequency analysis of the EEG signals. The choice of the db4 wavelet is grounded in its ability to provide a good balance between time and frequency localization, which is particularly beneficial for analyzing non-stationary EEG signals that exhibit rapid changes. The db4 wavelet allows for effective representation of transient features in the EEG data, making it suitable for capturing brain activity associated with pain perception.

#### 3.3.2. Statistical Analysis

Following the decomposition of EEG signals using discrete wavelet transform (DWT), we conducted a statistical analysis on the obtained wavelet coefficients to extract features indicative of pain perception.

For our statistical analysis, we employed both parametric and non-parametric statistical tests, as appropriate for the data characteristics. Specifically, we used parametric tests when the data met the assumptions of normality and homogeneity of variance, and non-parametric tests in cases where these assumptions were violated.

The selection of wavelet coefficients does not depend on normality and homogeneity of variance tests, as these assumptions are not required for our analysis. This flexibility allows us to accurately capture pain-relevant features without being constrained by strict statistical assumptions.

The analysis involved computing various statistical measures, including zero-crossing rate, percentiles, mean, median, standard deviation, variance, and root mean square. We have included effect sizes for the statistical models used to evaluate the method’s efficiency. In instances where effect size reporting was not feasible, we have provided justifications for these omissions.

We employed statistical feature extraction from the wavelet coefficients derived using DWT, as detailed in the pseudo-code (Algorithm 1). These extracted features were specifically designed to capture pain-relevant information from the EEG signals.
**Algorithm 1 **Feature Extraction Process**Input**
    EEG            EEG Dataset
**Output**
         X            Features matrix        $Y_{2}$            Two-class Label        $Y_{3}$            Three-class Label**for all** i∈ **EEG do**▹ for each Event    **event**, event_name ← **event_annotation** (EEG)
    **for all** epoch ∈ **Event do**▹ for each Epoch        **raw** ← **Get_Epoch_raw_EEG_data** (epoch)▹ Data Segmentation        **Y** ← event_name
        **for all** j ∈ **raw do**▹ for each raw datum           **List_coeff** ← **wavedec** (j, db4, level = 5)▹ Wavelet transform           **S** ← **calculate_statistics** (List_coeff)▹ Calculate into a feature vector           **X** ← **S**▹ Combine feature vectors for each coefficient        **end for**
    **end for**
**end for**
$Y_{2}$, $Y_{3}$ ← **label_Mapping** (Y)▹ Class labeling

After the feature extraction stage, a list of features was generated from the EEG signals. This list served as the input for subsequent classification stages. The features derived from the statistical analysis of the wavelet components obtained through DWT provided valuable insights into the inherent characteristics of the EEG signals related to pain perception.

### 3.4. Data Augmentation

Limited data availability is a persistent challenge in EEG-based pain detection. To address this limitation, this section explores the application of data augmentation techniques. Data augmentation aims to artificially expand training data by generating new synthetic data points derived from existing samples [25].

This study investigates the potential of two established data augmentation techniques commonly employed with EEG signals: data transformation and balancing. Freer and Yang [25] demonstrated the effectiveness of data transformation techniques in EEG classification. Similarly, a data balancing approach called the SMOTE [26] has been demonstrated to improve performance by addressing class imbalance in EEG datasets.

The data augmentation techniques were applied to the training dataset after the data were split to ensure that the test set remained untouched and representative of the original data distribution. This approach allows us to enhance the training data without introducing bias into the evaluation metrics. The specific augmentation techniques employed include noise injection and frequency modulation, which serve to increase the variability of the training samples and improve the model’s robustness.

The following sections detail the specific implementation of these data augmentation techniques within the context of the proposed pain detection system.

#### 3.4.1. Data Transformation

This study applies several data augmentation techniques to expand the training dataset for EEG-based pain detection. Three primary techniques were employed: data multiplication, data noise injection, and frequency modulation.

Data Multiplication: To introduce variations in existing samples, the training data were multiplied by a small factor denoted by the constant ($C_{mult}$ = 0.05). This process is mathematically represented in Equation (1), where data[i] refer to data from a control class [25]. Two versions were created for each sample: one obtained by multiplying the data by (1 + $C_{multi}$) and another with division by multiplying the data by (1 − $C_{multi}$). Both versions, along with their corresponding labels, were added to the training data.

(1) $$data_{multi1,multi2}=data\left[i\right]*\left(1_{-}^{+}C_{multi}\right)$$

Data Noise Injection: Uniformly distributed random noise was introduced into the training data to improve variability. The noisy data samples, along with their corresponding labels, were subsequently appended to the original dataset. The noise standard deviation was set to 2% of the standard deviation of the training data. Equation (2) demonstrates the noise addition process, where data[i] represents data from a control class, σ denotes the standard deviation, and $C_{noise}$ denotes a constant value; rand is a random number uniformly distributed between −0.5 and 0.5; this range was chosen to introduce controlled variability into the dataset without significantly altering the underlying signal characteristics [25].

(2) $$data_{noise}=data\left[i\right]+rand*\frac{\sigma (data[i])}{C_{noise}}$$

Frequency Modulation: The frequency content of the training data was modified by shifting its frequency spectrum. Two variations of each input sample were generated: one with a negative shift and another with a positive shift [25]. The modulation process is expressed by Equation (3):

(3) $$data_{freq1,freq2}=F_{shift}\left(data\left[i\right]{,}_{-}^{+}C_{freq}\right)$$

where $C_{freq}$ denotes the maximum control class and $F_{shift}$ denotes a function that performs frequency shifting using the Hilbert transform. The mathematical definition of $F_{shift}$ is provided in the following equation for further clarity [25].

(4) $$F_{shift}\left(d,C\right)=F^{-1}\left(F\left(d\right)2U\right)*e^{2j\pi *C*d_{t}}$$

where *d* represents the data, *C* represents a control class of the data, *F* represents the Fourier transform, *U* represents the unit step function, and $d_{t}$ denotes the reciprocal of the data collection frequency [25]. The frequency shift factor was set to 0.2 based on preliminary experiments that indicated this value effectively balanced the frequency modulation without introducing excessive distortion to the EEG signals. The frequency shift factor value was set after conducting a series of tests using various values ranging from 0.1 to 0.4. The other parameter $\left(C_{m}ult,C_{f}req,C_{n}oise\right)$ values were determined through preliminary experiments evaluating different modulation levels and selecting the configuration that yielded the best model accuracy. When multiplied by 2, 2*U* allows for a defined frequency shift that activates at certain points in the signal processing.

#### 3.4.2. Data Balancing

We employed the SMOTE to address the potential bias introduced by class imbalance in pain detection training dataset. Class imbalance occurs when one class, typically the pain-free class, has significantly fewer samples than the pain class. This imbalance can lead to biased model performance that favors the majority class. The SMOTE identifies the k nearest neighbors for each minority class sample and generates synthetic samples by interpolating between the selected sample and its neighbors in the feature space. This approach effectively increases the representation of minority classes, resulting in a more balanced dataset for training the classifier and potentially improving model generalizability across both pain and pain-free states [26].

Before balancing the 2-level class, the training dataset comprised two classes with varying sample sizes: pain (1152), and no pain (220). After applying the SMOTE, each class contained a balanced representation of 1152 samples. Before balancing the 3-level class, the dataset comprised three classes with varying sample sizes: high (108), moderate (864), and low (1135). After applying the SMOTE, each class contained a balanced representation of 1135 samples.

**Figure 7:**
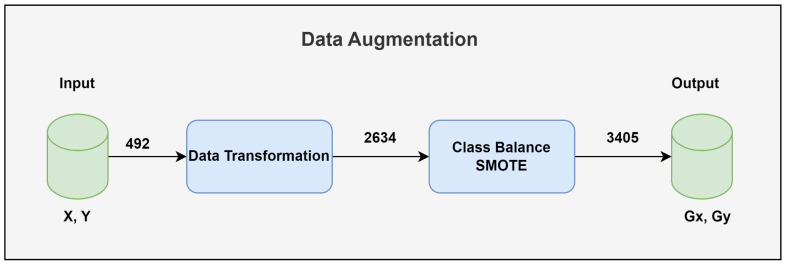
Data augmentation.

Figure 7 shows the significant increase in training data size achieved via data augmentation. The dataset size increased from initial 492 samples to 2634 samples after applying the data transformation techniques. In addition, the class imbalance issue was addressed for binary and ternary classification.

### 3.5. Classification

This section describes the classification implementation to categorize pain into two and three severity levels. By leveraging both ML and DL techniques, we investigate the effectiveness of various classification models in pain detection. As baseline models, we use support vector machine (SVM), random forest (RF), and k-nearest neighbor (KNN) classifiers. In addition, we explore the application of deep neural networks for pain detection, focusing specifically on CNNs and RNNs. These architectures are promising for capturing the intricate patterns and temporal dependencies inherent in pain data.

#### 3.5.1. Machine Learning

Several ML techniques, including SVM, RF, and KNN, were implemented using the scikit-learn library in Python.

First, we applied the SVM classifier without any regularization technique using the default hyperparameters. The SVM classifier was implemented with the default setup, including the rbf (radial basis function) kernel and default parameters of C = 1.0 and gamma = “scale”.

Next, we employed the KNN classifier, which is a non-parametric algorithm that classifies samples based on their nearest neighbors. The KNN classifier was implemented with the number of neighbors (K = 3) and the Euclidean distance metric.

In addition, we employed the RF classifier, which is an ensemble learning method that combines multiple decision trees to make predictions. Each decision tree in the RF independently classifies the input data. The final classification is determined by aggregating the results of all trees. The RF classifier was implemented with the number of trees in the forest = 100 and the Gini criterion for splitting.

#### 3.5.2. Deep Learning

This section explores the application of DL techniques, specifically deep neural networks, for pain detection using EEG signals. We investigate the potential of two prominent neural network architectures: CNNs and RNNs.

**Figure 8:**
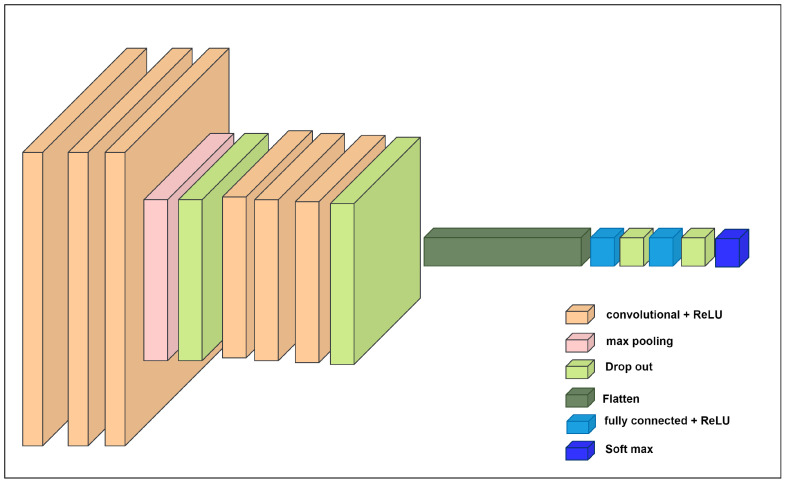
CNN architecture.

**CNN Model:** We propose a CNN architecture (Figure 8) specifically designed to extract pain-related features from EEG data. This figure provides an overview of the different multiple layers in the CNN model, which comprises convolutional, pooling, dropout, and fully connected layers. The convolutional layers capture the spatial patterns within the signals. Then, pooling layers are employed to reduce data dimensionality and enhance robustness against noise. Dropout layers are strategically incorporated to prevent overfitting during training. Finally, fully connected layers perform high-level reasoning and classification to differentiate between pain and nonpain states.

The convolutional layers employ the rectified linear unit (ReLU) activation function. To mitigate overfitting, dropout layers with a rate of 0.25 are incorporated after each pooling layer. After the convolutional and pooling stages, the extracted feature maps are flattened into a one-dimensional vector, which serves as the input to the fully connected layers. These fully connected layers comprise two hidden layers that also use the ReLU activation function. Dropout layers with rates of 0.5 and 0.3 are applied after each hidden fully connected layer. Finally, the output dimension of the last fully connected layer depends on the number of classes. In pain detection tasks, the number is set to 2, whereas, in pain severity classification, it is set to 3.

The CNN model was trained using the Adam optimizer at a learning rate of 0.00009. Categorical cross-entropy was employed as the loss function. The training process involved 100 epochs. To evaluate model performance, accuracy and root mean square error (RMSE) metrics were used.

Hyperparameter optimization was performed using grid search, a technique that systematically evaluates a predefined grid of hyperparameter values to determine the combination that yields the best model performance [27]. In this study, a grid search was employed to optimize the dropout rate, learning rate, and number of epochs. The GridSearchCV function from scikit-learn was used with the KerasClassifier wrapper for integration with the DL model.

A grid search parameter space (param_grid) was defined to explore a range of values for each hyperparameter:

- Dropout rate: A range of 0.0–0.9 was investigated to assess the impact of dropout on overfitting prevention.
- Learning rate: A set of learning rates (0.2, 0.5, 0.05, 0.005, 0.009, 0.0009, 0.0009, and 0.00009) was evaluated to determine the optimal learning speed for the model.
- Epochs: The number of training iterations was varied (50, 80, 100, 120, and 200) to determine the appropriate training duration.

The GridSearchCV function then fits the model with these hyperparameter combinations using the training data. The resulting model with the best performance on the validation set was selected to provide the optimal hyperparameters for the proposed CNN model.

**Figure 9:**
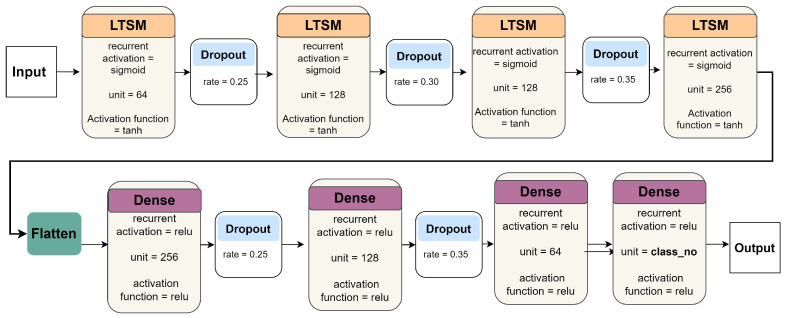
RNN architecture.

**RNN:** This study employs a RNN model for pain detection using EEG signals (Figure 9). The experiment leveraged the Keras DL library to construct a multilayer RNN architecture. The model employed a stack of LSTM layers, each with a predefined number of units and a tanh activation function. These LSTM layers were specifically designed to capture the temporal dependencies in the EEG data. To prevent overfitting, dropout layers were strategically placed after each LSTM layer. In addition, the model incorporated fully connected layers equipped with various activation functions, such as ReLU, to map the extracted features to pain classes.

Finally, the fully connected layers used the softmax activation function to generate class probabilities. The model was compiled using the Adam optimizer, a categorical cross-entropy loss function, and metrics such as accuracy and RMSE. The training process included 100 and 50 epochs for pain/no-pain detection and three-level pain severity classification, respectively. Validation data were employed to evaluate model performance during training.

The model architecture and its hyperparameters were determined through an iterative process of empirical analysis and experimentation that involved systematically evaluating factors such as the number of layers, number of units per layer, activation functions, and overall model complexity. By iteratively training and evaluating the model, we fine-tuned these parameters and optimized the model performance.

Our experimentation revealed a relationship between the number of units in the LSTM layers and model performance. An increase in the number of units within each LSTM layer, followed by a corresponding decrease in the number of units within the fully connected layers, yielded a more effective model architecture.

However, model performance was compromised when the number of units deviated significantly from the range of 32–512 units. In these instances, the ability of the model to learn and capture intricate patterns and representations was restricted. The identified range (between 64 and 256 units) appeared to achieve an optimal balance between model complexity and computational efficiency, thereby facilitating superior representation learning and improving the predictive capabilities of the model.

An iterative hyperparameter tuning strategy was employed to select activation functions for the RNN model. This approach involved the systematic evaluation of various activation functions for both the LSTM and fully connected layers. The experiment also had the model run iteratively, with each iteration employing a different activation function, such as ReLU, sigmoid, and tanh.

The evaluation process examined the LSTM layers and then the fully connected layers while maintaining the softmax activation function in the final layer. This systematic approach facilitated the identification of the activation functions that yielded optimal performance in terms of accuracy and overall model efficacy. The iterative nature of this process allows fine-tuning and optimizing activation functions specific to the LSTM model.

## 4. Results

This section presents the results of the proposed EEG-based pain detection system. The analysis focuses on the prediction of pain outcomes using two types of labeling: pain/no-pain detection and pain severity classification. In the pain detection formulation, labels were categorized as “no pain” and “pain”. For the pain severity classification formulation, the labels were assigned as “low”, “moderate”, and “high pain”.

The experiments involved the evaluation of various classifiers, including SVM, RF, KNN, CNN, and RNN, on datasets with different numbers of classes, namely, pain/no-pain detection and pain severity classification. To evaluate the performance of these classifiers, various evaluation metrics were calculated such as recall, accuracy, precision, recall, and F1-measure. To assess the model generalizability and prevent overfitting, we employed holdout and 10-fold cross-validation techniques. Holdout is a simple approach to partitioning a dataset into training and testing subsets, because it allows for a quick evaluation of the model performance [28]. In this study, we divided the dataset into training and test sets using an 80:20 split. To ensure the reliability of our pain classification results, we employed a k-fold cross-validation approach, which offers a robust estimate of the model performance by iteratively training and testing the models on different subsets of the data, reducing the potential bias inherent in a single holdout split [28]. K-fold cross-validation involves randomly partitioning a dataset into k equal-size subsets, training the models on k-1 subsets, and testing on the remaining 1 subset. Then, this process is repeated k times [27]. In this study, we set k to 10.

### 4.1. Pain/No-Pain Detection

This section evaluates the performance of several ML and DL classifiers for pain detection (no pain vs. pain) using holdout and 10-fold cross-validation techniques.

The evaluation metrics of all classifiers are presented in Table 2. This evaluation provides valuable insights into the strengths and limitations of each classifier relative to accurately distinguishing between the pain and no-pain conditions.

In the holdout cross-validation, the CNN achieved the highest accuracy (90.69%) among all models, outperforming the baseline ML approaches (SVM, KNN, and RF). Furthermore, the CNN exhibited strong precision, recall, and F1-measure scores (all of which were approximately 90.69%), demonstrating its effectiveness in extracting relevant pain-related features and patterns, and facilitating accurate predictions. The RNN demonstrated remarkable performance with an accuracy of 89.82% and consistently high scores in precision, recall, and F1-measure.

To further validate the consistency of the DL models, we also performed 10-fold cross-validation. The results demonstrate that the RNN model outperformed the other models, attaining an accuracy of 91.84%. The CNN model also achieved high performance with an accuracy of 89.74%. These results highlight the ability of DL to model the temporal dynamics of pain detection data, which is crucial for accurate classification.

Among the ML classifiers, the KNN classifier achieved accuracy rates of 91.61% and 79.69% in 10-fold and holdout cross-validation, respectively. Although exhibiting slightly lower performance than the CNN model in terms of holdout cross-validation and the RNN model in terms of 10-fold cross-validation, the KNN classifier exhibited reasonable accuracy and precision (79.36% and 89.90%, respectively) in pain detection.

The RF model achieved an accuracy of 88.08%, outperforming the KNN in holdout cross-validation but falling short of the CNN. The RF model exhibited high precision (88%) and recall (87%) scores, indicating its capability to accurately detect pain and categorize it as either “pain” or “no pain”. The F1-measure score (87.31%) further supports the RF model’s overall performance.

Similarly, the SVM achieved accuracy rates of 82.55% and 84.21% in holdout and 10-fold cross-validation, respectively, exhibiting lower accuracy than the other models. However, this performance serves as a baseline for gaining insights into the abilities of the SVM model. The SVM exhibited a precision of 82.55%, indicating a moderate success rate in identifying pain instances it classified. However, its recall score of 86.75% suggests that it is unable to accurately classify all actual high-pain instances. The F1-measure (82.52%) reflects a trade-off between precision and recall. These scores suggest that the SVM better capture the complexities within the pain intensity data compared to other ML and DL approaches.

**Figure 10:**
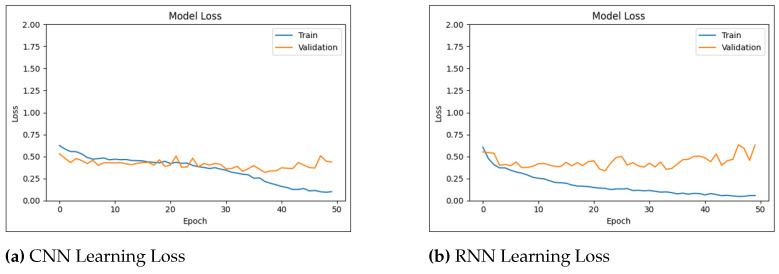
Learning accuracy and loss for pain/no-pain detector.

Figure 10 shows the learning curves of the CNN and RNN models over 25 epochs. The learning curves visualize the improvement in model performance with exposure to training data. These curves are essential for evaluating model convergence and identifying potential problems associated with overfitting or underfitting.

### 4.2. Pain Severity Classification

The performance of various classifiers, including SVM, RF, KNN, CNN, and RNN, in categorizing three-level pain severity was evaluated using multiple metrics with the holdout and 10-fold cross-validation techniques. The evaluation results for the classifiers are presented in Table 3.

The DL classifiers (CNNs and RNNs) achieved superior performance compared to the baseline classifiers. The RNN model exhibited the highest accuracy of 86.71%, and the precision, recall, and F1-measure scores reached 86.71% in holdout cross-validation. The CNN model exhibited comparable performance, with an accuracy of 87.94% in 10-fold cross-validation and consistent scores across the remaining evaluation metrics. These findings suggest the effectiveness of DL models in capturing relevant features and patterns associated with pain severity, which facilitates accurate pain detection.

In contrast, the performance of the baseline ML classifiers varied. The KNN classifier achieved an accuracy of 65.08% in holdout validation, which is significantly lower than that of the DL models. In addition, the KNN model exhibited a precision score of 65.08%, recall of 66.40%, and F1-measure of 41.58%, indicating its limitations in accurately classifying pain severity levels. The RF classifier outperformed the KNN model, achieving an accuracy of 75.90%, and it demonstrated balanced precision, recall, and F1-measure scores of approximately 75%. Furthermore, the RF model achieved an accuracy of 79.01% in cross-validation, indicating its ability to classify pain severity across all categories (low, moderate, and high) with some success. The SVM classifier achieved the lowest accuracy among the baseline models in holdout cross-validation (56.54%), with a precision of 65.54%, recall of 34.98%, and F1-measure of 41.58%.

**Figure 11:**
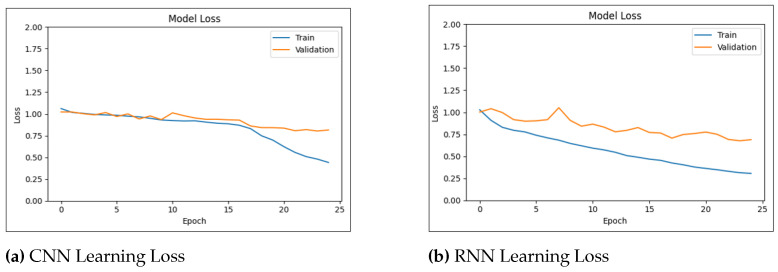
Learning accuracy and loss for three-level pain classification.

Figure 11 shows the learning curves of the CNN and RNN models over 25 epochs in three-level pain severity classification.

This investigation revealed the superiority of DL classifiers compared with conventional ML approaches (SVM, KNN, RF) for three-level pain severity categorization (low, moderate, high) based on accuracy and F1-measure. Notably, the RNN model demonstrated the exceptional performance of DL models, indicating their potential to provide robust and reliable pain severity classification. These findings have significant implications for developing pain assessment tools in healthcare and clinical settings.

## 5. Discussion

**Figure 12:**
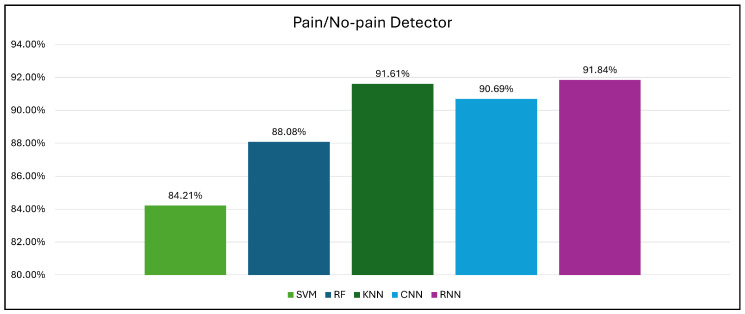
Pain/no-pain detector results.

**Figure 13:**
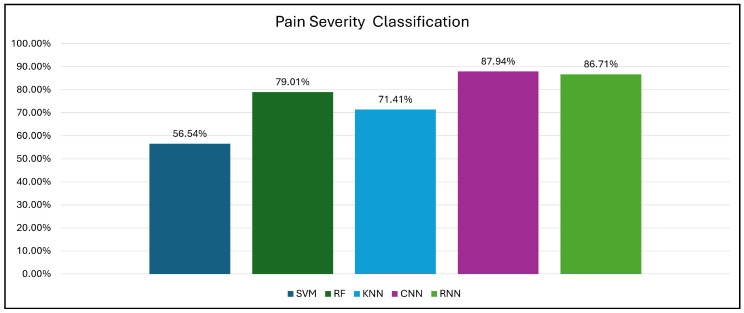
Pain severity classification results.

The conducted experiments indicate that the DL models (CNNs and RNNs) achieved the most promising results. The CNN model was initially trained and evaluated on datasets with binary (Figure 12 and ternary pain classification labels (Figure 13). This resulted in accuracy rates of 87.94% and 90.69% for three-level pain classification and pain/no-pain detection, respectively. Similarly, the RNN model achieved accuracy rates of 86.71% and 91.84% for the three-level pain classification and pain/no-pain detection, respectively.

Our initial exploration of the baseline ML algorithms for the pain/no-pain detection and pain severity classification tasks demonstrated that the RF classifier achieved the highest accuracy (88.08% and 79.01%) in the pain detection and classification tasks, respectively. The initial result establishes the baseline performance for subsequent evaluations. The strength of the RF model lies in its ensemble of decision trees, which enables it to handle complex datasets and capture nonlinear relationships, typically leading to competitive performance [27].

The KNN classifier also demonstrated promising results in the pain detection task, achieving an accuracy of 91.61%. This finding suggests that the KNN model is effective for accurately classifying pain into two distinct categories. However, the performance of the KNN model is highly sensitive to the number of neighbors (k) and distance metrics. This sensitivity is evident in the three-class classification task, where the accuracy of the KNN model decreases to 71.41%.

The DL model demonstrates significant potential for effectively classifying pain severity and detecting pain/no-pain states. The CNN model achieved accuracy rates of 90.69% and 87.94% for pain/no-pain detection and pain severity classification, respectively. Conversely, the RNN model achieved accuracy rates of 91.84% and 86.71% in pain/no-pain detection and pain severity classification, respectively.

The outcomes of this study illustrate the effectiveness of the DL CNN and RNN models in contrast to the conventional ML classifiers SVM, KNN, and RF for both pain detection and severity classification tasks. This advantage is attributed to the inherent ability of DL models to extract complex representations and underlying patterns from data. This ability translates into superior performance and accuracy when predicting high- and low-pain levels.

### 5.1. Impact of Data Augmentation

**Figure 14:**
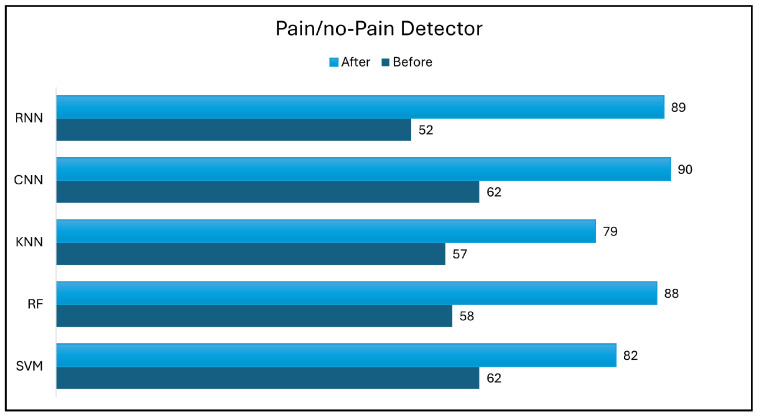
Data transformation results for pain/no-pain detector.

**Figure 15:**
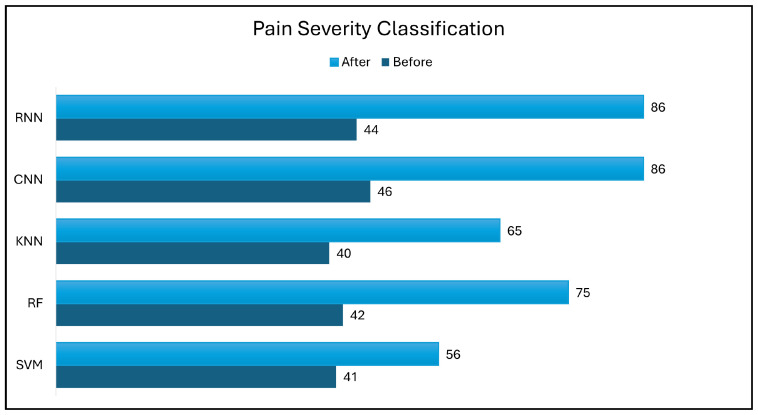
Data transformation results for pain severity classification.

Figure 14 and Figure 15 show the impact of data transformation, which is a data augmentation technique, on the performance of various classifiers using holdout evaluation. The accuracy of the classifiers was evaluated before and after data augmentation to assess its effectiveness, ensuring that the augmentation preserved the physiological relevance of the original data.

Prior to data augmentation, all classifiers exhibited modest accuracy, with the CNN model achieving the highest performance at 62% and 46% in pain/no-pain detection and pain severity classification, respectively. However, these accuracy levels were relatively low compared to the performance of the other pain detection methods.

Data transformation yielded significant improvements in classification accuracy across all classifiers for both pain/no-pain detection and pain severity classification tasks. For pain/no-pain detection, SVM accuracy increased by 20% to 82% after data transformation. Similarly, the RF model achieved an accuracy of 88%, which is a 33% improvement. The KNN model also exhibited a notable accuracy increase of 22%, which eventually reached an accuracy of 79%. The CNN classifier exhibited accuracy improvement, with its accuracy reaching 90% after data transformation, representing a 28% increase. The RNN classifier demonstrated the most significant improvement, achieving an accuracy of 89% after data transformation, which represents a 37% increase.

In the pain severity classification task, the SVM accuracy increased by 15% to 56% after data transformation. Similarly, the RF model achieved 75% accuracy, which represents a 33% improvement. The KNN classifier also experienced a significant accuracy increase of 15%, eventually reaching 65% accuracy. Notably, the CNN classifier exhibited the most significant improvement, with its accuracy reaching 83% after data transformation, indicating a 37% increase. This significant improvement in CNN accuracy can be attributed to the inherent capacity of CNNs to extract complex features. It may also have been influenced by the requirement for larger training datasets. The RNN classifier exhibited a notable improvement, achieving an accuracy of 79% after data transformation, indicating a 35% increase. The considerable improvement in RNN accuracy is likely due to its intrinsic capability for intricate feature extraction.

Recent advances in ML and DL have highlighted the importance of data transformation techniques. One such technique, data balancing, has emerged as a crucial step in data augmentation. This study investigates the impact of the SMOTE on classifier performance. The SMOTE was designed to oversample minority classes in datasets, resulting in a more balanced class distribution. In our DL task involving three initial classes, the CNN model achieved an accuracy of 83% before applying the SMOTE. Following SMOTE balancing, CNN accuracy increased to 86%, which represents a significant improvement of 3%. In addition, the RNN model achieved the highest increase after applying the SMOTE, achieving an accuracy of 86%, which represents a 7% improvement. This improvement can be attributed to the balanced class representation, which enables the CNN and RNN models to learn from an equal number of samples per class, leading to more accurate predictions.

While these results indicate that data augmentation can enhance classifier performance, we recognize the potential risk of overfitting due to the synthetic nature of augmented samples. To mitigate this risk, we employed cross-validation techniques to ensure that our models generalize well to unseen data.

In addition, we explored the binary pain detection task to assess the influence of data balancing on DL performance. Before applying the SMOTE, the CNN model achieved an accuracy of 90.09%. After applying the SMOTE to balance the two classes, accuracy remained high (90.69%), representing a minor increase of 60%. This suggests that the initial class distribution for the binary case was relatively balanced, and the application of the SMOTE had a negligible effect on the overall accuracy.

### 5.2. Comparison with Related Works

In this subsection, a comparative analysis was performed between the results of this study and previous studies on pain classification. The pain classification results of this study are in good agreement with those of previous studies that adopted the same EEG dataset for the same classification purposes. The pain classification results of this study demonstrate good performance in the pain/no-pain detection task, differentiating between pain and no-pain conditions, with a high classification accuracy of 91.84%.

Table 4 compares the results of this study and existing pain classification studies. In addition, even for the pain severity classification task, differentiating between low-, moderate-, and high-pain conditions, our model maintained a strong accuracy of 87.94%. This indicates that the feature engineering, model optimization, and DL methods employed in this study improve performance across pain detection and classification compared to earlier studies. This comparative analysis highlights the progress made in this study and its valuable contributions to the field of brain-based pain detection. The existing literature on EEG-based pain classification has explored various methodological approaches that encompass various pain classes and employ ML and DL techniques. Several studies have focused on binary classification, distinguishing between no-pain and pain states, with accuracy rates ranging from 72.7% to 84% for ML model utilization [18,19,29,30] and 73% to 87% for the DL technique [21,31].

In contrast, this study demonstrates advances in the performance of EEG-based pain classification. For pain/no-pain detection, the RNN model obtained an accuracy of 91.84%. This model outperformed the conventional ML classifiers, with the highest accuracy among the SVM, RF, and KNN models reaching 91.69%. In addition, for pain severity classification (low, moderate, and high), the CNN model obtained an accuracy of 87.94%, whereas the RNN model obtained an accuracy of 86.71%. This outperformed the conventional ML classifiers, with the highest accuracy among the SVM, RF, and KNN models reaching 79.01%.

The comparison analysis focused on previous studies classifying pain in healthy patients rather than those with chronic pain. The rationale for this distinction is that studies on chronic pain classification typically examine how patients interact with and respond to pain in their daily lives, which introduces additional complexities compared to assessing acute pain responses in healthy individuals.

The performance of the DL models in the pain/no-pain detection and pain severity classification tasks presented in this study aligns well with the emerging body of research on the application of DL techniques to EEG-based pain assessment. Recent studies have demonstrated the promising capabilities of DL models to extract salient features from EEG data with high accuracy when discriminating between pain and no-pain states and classifying different pain severity levels.

The findings of this study, combined with the advances reported in the recent literature, highlight the growing momentum and value of DL techniques in the field of EEG-based pain assessment. The consistent high performance of the CNN and RNN models across the two tasks highlights their ability to effectively leverage the rich information in EEG signals to develop robust and accurate pain detection and severity classification systems.

## 6. Conclusions

In this study, a BCI system was developed to investigate the feasibility of using EEG for pain detection and classification. We conducted rigorous analyses to investigate DL, particularly CNNs and RNNs, in pain classification. In addition, we compared the classification performance of the proposed DL-based approaches with that of conventional ML classifiers, such as RF, SVM, and KNN. The comprehensive data analysis results revealed that the DL-based classifier methods, which leverage the inherent capabilities of deep neural networks for pattern recognition, demonstrated superior performance compared to the ML-based classifiers in accurately classifying pain states.

The findings of this study on pain classification align with previous research that highlighted the potential of EEG-based approaches, showing significant improvements in classification accuracy. In clinical practice, the proposed BCI system could assist healthcare providers in monitoring and assessing pain more effectively, potentially leading to improved patient outcomes.

These results indicate several promising directions for future research. First, the creation of shared, publicly available datasets would facilitate cross-validation and collaboration, thereby enhancing the understanding and assessment of pain. Second, further exploration of feature engineering strategies is essential, as this study utilized specific techniques to extract informative features from high-dimensional neuroimaging data. Lastly, future studies should investigate diverse methods for feature extraction, encompassing time-, frequency-, and time–frequency-domain features, along with advanced selection and dimensionality reduction techniques. This comprehensive approach could lead to more robust and accurate pain detection algorithms.

While this study demonstrated promising results, it is essential to acknowledge limitations, such as the need for larger, diverse datasets to validate the findings and ensure generalizability across different populations. We recognize that relying solely on EEG signals may not capture the full complexity of pain perception, as pain is influenced by a multitude of physiological and psychological factors. To achieve a more comprehensive understanding of pain, it is crucial to incorporate additional physiological measurements, such as fMRI, heart rate variability, Galvanic Skin Response (GSR) signals, and behavioral assessments. We recommend that future studies integrate these diverse data sources, which could significantly enhance pain assessment methodologies and contribute to a more nuanced understanding of pain mechanisms. 

## Figures and Tables

**Table 1:** Pain dataset conditions and related pain measurements.

Condition	Pain Measurement
Perception condition	Self-reported pain rating: participants rated pain severity on a scale of 0–109 after varying levels of painful laser stimuli (low, medium, high) presented every 8–12 s.
Motor condition	Participants’ reaction time to pressing a button after each laser stimulus.
Autonomic condition	Participants’ recordings of skin conductance responses.
Combined condition	Participants reacted to the laser stimuli in three different ways: pain rating, reaction times, and SCRs.

**Table 2:** Pain/no-pain detector performance metrics.

CV	Classifier	Accuracy	Precision	Recall	F1
Holdout	SVM	82.55%	82.55%	82.67%	82.52%
	RF	88.08%	88.08%	87.40%	87.31%
	KNN	79.36%	79.36%	89.90%	81.37%
	CNN	90.69%	90.69%	90.69%	90.69%
	RNN	89.82%	89.82%	89.82%	89.82%
10-Fold	SVM	84.21%	84.05%	100%	91.27%
	RF	86.60%	86.25%	99.79%	92.47%
	KNN	91.61%	94.85%	95.02%	94.92%
	CNN	89.74%	89.69%	99.16%	94.17%
	RNN	91.84%	91.35%	99.65%	95.31%

**Table 3:** Pain severity classification performance metrics.

CV	Classifier	Accuracy	Precision	Recall	F1
Holdout	SVM	56.54%	56.45%	34.98%	41.58%
	RF	75.90%	75.90%	75.95%	74.66%
	KNN	65.08%	65.08%	66.40%	65.47%
	CNN	86.52%	86.52%	86.52%	86.52%
	RNN	86.71%	86.71%	86.71%	86.71%
10-fold	SVM	54.78%	30.51%	36.73%	28.78%
	RF	79.01%	69.01%	57.42%	58.41%
	KNN	71.41%	63.66%	63.08%	62.97%
	CNN	87.94%	88.66%	98.18%	93.15%
	RNN	82.96%	84.53%	65.30%	68.79%

**Table 4:** Comparison of EEG-based pain classification approaches from recent literature.

Reference	Classes of Pain	Classifier	Accuracy
Nezam et al., 2018 [29]	5 (no pain, 1st–4th level)	KNN and SVM	83%
Bonotis et al., 2019 [30]	5 (no, low, mid, high, unbearable)	SF	72.7%
Alazrai et al., 2019 [18]	2 (no pain and pain)	SVM	83%
Sai et al., 2019 [19]	2 (no pain and pain)	SVM	84%
Wang et al., 2020 [31]	2 (high and low pain)	Auto-encoder and LR	74%
Chen et al., 2022 [21]	2 (no pain and pain)	CNN	83%
Fu et al., 2024 [32]	2 (pain and no pain)	Transformer and CNN	87.83%
This study	2 (no pain and pain)	SVM, KNN, and RF	91.69%
	3 (low, moderate, and high pain)	SVM, KNN, and RF	79.01%
	2 (no pain and pain)	CNN and RNN	91.84%
	3 (low, moderate, and high pain)	CNN and RNN	87.94%

## Data Availability

The original data presented in the study are openly available in OSF “Brain mediators of pain” at https://osf.io/bsv86/ (accessed on 20 January 2024).

## References

[1] Herr K., Coyne P.J., McCaffery M., Manworren R., Merkel S. (2011). Pain assessment in the patient unable to self-report: Position statement with clinical practice recommendations. Pain Manag. Nurs..

[2] Gao X., Wang Y., Chen X., Gao S. (2021). Interface, interaction, and intelligence in generalized brain–computer interfaces. Trends Cogn. Sci..

[3] Mridha M.F., Das S.C., Kabir M.M., Lima A.A., Islam M.R., Watanobe Y. (2021). Brain-computer interface: Advancement and challenges. Sensors.

[4] Panoulas K.J., Hadjileontiadis L.J., Panas S.M. (2010). Brain-computer interface (BCI): Types, processing perspectives and applications. Multimedia Services in Intelligent Environments: Integrated Systems.

[5] Ghosh R. (2023). A Survey of Brain Computer Interface Using Non-Invasive Methods. arXiv.

[6] Sitaram R., Caria A., Veit R., Gaber T., Rota G., Kuebler A., Birbaumer N. (2007). FMRI brain-computer interface: A tool for neuroscientific research and treatment. Comput. Intell. Neurosci..

[7] Alshehri H., Al-Nafjan A., Aldayel M. (2025). Decoding Pain: A Comprehensive Review of Computational Intelligence Methods in Electroencephalography-Based Brain–Computer Interfaces. Diagnostics.

[8] Barnova K., Mikolasova M., Kahankova R.V., Jaros R., Kawala-Sterniuk A., Snasel V., Mirjalili S., Pelc M., Martinek R. (2023). Implementation of artificial intelligence and machine learning-based methods in brain-computer interaction. Comput. Biol. Med..

[9] Cascella M., Schiavo D., Cuomo A., Ottaiano A., Perri F., Patrone R., Migliarelli S., Bignami E.G., Vittori A., Cutugno F. (2023). Artificial intelligence for automatic pain assessment: Research methods and perspectives. Pain Res. Manag..

[10] Chollet F. (2021). Deep Learning with Python.

[11] Zhang X., Yao L., Wang X., Monaghan J., Mcalpine D., Zhang Y. (2019). A survey on deep learning based brain computer interface: Recent advances and new frontiers. arXiv.

[12] Zhang A., Lipton Z.C., Li M., Smola A.J. (2021). Dive into deep learning. arXiv.

[13] Tiemann L., Hohn V., Ploner M. (2018). Brain Mediators of Pain. https://osf.io/BSV86/.

[14] Dworkin R.H., Turk D.C., Farrar J.T., Haythornthwaite J.A., Jensen M.P., Katz N.P., Kerns R.D., Stucki G., Allen R.R., Bellamy N. (2005). Core outcome measures for chronic pain clinical trials: IMMPACT recommendations. Pain.

[15] Melzack R. (1999). Pain—An overview. Acta Anaesthesiol. Scand..

[16] Tiemann L., Hohn V.D., Ta Dinh S., May E.S., Nickel M.M., Gross J., Ploner M. (2018). Distinct patterns of brain activity mediate perceptual and motor and autonomic responses to noxious stimuli. Nat. Commun..

[17] Traylor Z. (2023). Sex Differences in Pain Perception and Event Related Neural Correlates of Pain.

[18] Alazrai R., Al-Rawi S., Daoud M.I. A Time-Frequency Distribution Based Approach for Detecting Tonic Cold Pain using EEG Signals. Proceedings of the 2019 IEEE 19th International Conference on Bioinformatics and Bioengineering (BIBE).

[19] Sai C.Y., Mokhtar N., Yip H.W., Bak L.L.M., Hasan M.S., Arof H., Cumming P., Mat Adenan N.A. (2019). Objective identification of pain due to uterine contraction during the first stage of labour using continuous EEG signals and SVM. Sādhanā.

[20] Elsayed M., Sim K.S., Tan S.C. (2020). A novel approach to objectively quantify the subjective perception of pain through electroencephalogram signal analysis. IEEE Access.

[21] Chen D., Zhang H., Kavitha P.T., Loy F.L., Ng S.H., Wang C., Phua K.S., Tjan S.Y., Yang S.Y., Guan C. (2022). Scalp EEG-based pain detection using convolutional neural network. IEEE Trans. Neural Syst. Rehabil. Eng..

[22] Vijayakumar V., Case M., Shirinpour S., He B. (2017). Quantifying and characterizing tonic thermal pain across subjects from EEG data using random forest models. IEEE Trans. Biomed. Eng..

[23] Alazrai R., Al-Rawi S., Alwanni H., Daoud M.I. (2019). Tonic cold pain detection using Choi–Williams time-frequency distribution analysis of EEG signals: A feasibility study. Appl. Sci..

[24] Afrasiabi S., Boostani R., Masnadi-Shirazi M.A., Nezam T. (2021). An EEG based hierarchical classification strategy to differentiate five intensities of pain. Expert Syst. Appl..

[25] Freer D., Yang G.Z. (2020). Data augmentation for self-paced motor imagery classification with C-LSTM. J. Neural Eng..

[26] Wardoyo R., Wirawan I.M.A., Pradipta I.G.A. (2022). Oversampling approach using radius-SMOTE for imbalance electroencephalography datasets. Emerg. Sci. J..

[27] Bonaccorso G. (2018). Machine Learning Algorithms: Popular Algorithms for Data Science and Machine Learning.

[28] Berrar D. (2019). Cross-Validation. Encyclopedia of Bioinformatics and Computational Biology.

[29] Nezam T., Boostani R., Abootalebi V., Rastegar K. (2018). A novel classification strategy to distinguish five levels of pain using the EEG signal features. IEEE Trans. Affect. Comput..

[30] Bonotis P.A., Tsouros D.C., Smyrlis P.N., Tzallas A.T., Giannakeas N., Glavas E., Tsipouras M.G. Automated assessment of pain intensity based on EEG signal analysis. Proceedings of the 2019 IEEE 19th International Conference on Bioinformatics and Bioengineering (BIBE).

[31] Wang J., Wei M., Zhang L., Huang G., Liang Z., Li L., Zhang Z. An autoencoder-based approach to predict subjective pain perception from high-density evoked EEG potentials. Proceedings of the 2020 42nd Annual International Conference of the IEEE Engineering in Medicine & Biology Society (EMBC).

[32] Fu Z., Zhu H., Zhang Y., Huan R., Chen S., Pan Y. (2024). A Spatiotemporal Deep Learning Framework for Scalp EEG-based Automated Pain Assessment in Children. IEEE Trans. Biomed. Eng..

